# Supplementary material for: Global hypo-methylation in a proportion of glioblastoma enriched for an astrocytic signature is associated with increased invasion and altered immune landscape
Source: eLife. 2022 Nov 22;11:e77335. doi: 10.7554/eLife.77335 (PMC9681209; doi:10.7554/eLife.77335)
Supplement: Figure 2—source data 1. [file elife-77335-fig2-data1.zip › Figure_2_source_data_1/Figure_2A/homerResults/motif3.similar.html]

motif3

## Information for motif3

A
C
T
G
A
T
C
G
A
G
C
T
A
G
T
C
G
A
T
C
A
G
T
C
T
C
A
G
A
G
T
C
A
G
T
C
C
A
G
T
A
G
T
C
G
T
A
C
  
Reverse Opposite:  

A
C
T
G
T
C
A
G
G
C
T
A
C
T
A
G
T
A
C
G
A
G
T
C
T
A
C
G
C
A
T
G
T
C
A
G
C
T
G
A
T
A
G
C
T
G
A
C
  

|  |  |
| --- | --- |
| p-value: | 1e-27 |
| log p-value: | -6.296e+01 |
| Information Content per bp: | 1.716 |
| Number of Target Sequences with motif | 32.0 |
| Percentage of Target Sequences with motif | 1.38% |
| Number of Background Sequences with motif | 3.8 |
| Percentage of Background Sequences with motif | 0.10% |
| Average Position of motif in Targets | 104.0 +/- 55.6bp |
| Average Position of motif in Background | 99.4 +/- 44.3bp |
| Strand Bias (log2 ratio + to - strand density) | 0.1 |
| Multiplicity (# of sites on avg that occur together) | 1.04 |
| Motif File: | file (matrix) reverse opposite |

### Similar de novo motifs found

|  |  |  |  |  |  |  |  |
| --- | --- | --- | --- | --- | --- | --- | --- |
| Rank | Match Score | Redundant Motif | P-value | log P-value | % of Targets | % of Background | Motif file |
| 1 | 0.655 | A G C T C G T A C T A G C G A T C A T G T A C G A T G C A C T G C T A G C A T G | 1e-16 | -38.694160 | 0.99% | 0.09% | motif file (matrix) |
| 2 | 0.618 | A G T C A C G T A G C T A G T C A T G C A C G T G A T C G A T C A C G T G A T C A G T C A G T C A G T C A G T C T A G C | 1e-12 | -29.072671 | 0.56% | 0.03% | motif file (matrix) |
| 3 | 0.671 | A C T G C G T A C G T A A C T G A C T G A C T G C G T A A C T G A C T G A G T C A C T G A C T G | 1e-5 | -11.796618 | 0.39% | 0.08% | motif file (matrix) |
